# Paneth cell-derived iNOS is required to maintain homeostasis in the intestinal stem cell niche

**DOI:** 10.1186/s12967-023-04744-w

**Published:** 2023-11-25

**Authors:** Lingxiao Huang, Zhenni Xu, Xudan Lei, Yujun Huang, Siyu Tu, Lu Xu, Jieying Xia, Dengqun Liu

**Affiliations:** 1 Radiation Oncology Key Laboratory of Sichuan Province, Department of Experimental Research, Sichuan Cancer Hospital & Institute, Sichuan Clinical Research Center for Cancer, Sichuan Cancer Center, Affiliated Cancer Hospital of University of Electronic Science and Technology of China, Chengdu, 610041 China; 2 School of Basic Medicine, Chengdu University of Traditional Chinese Medicine, Chengdu, 610075 China; 3 Animal Experiment Center of Sichuan Academy of Traditional Chinese Medicine Sciences, Chengdu, 610041 China

**Keywords:** iNOS, Paneth cell, Intestinal stem cell, Proliferation, Differentiation

## Abstract

**Background:**

Mammalian intestinal epithelium constantly undergoes rapid self-renewal and regeneration sustained by intestinal stem cells (ISCs) within crypts. Inducible nitric oxide synthase (iNOS) is an important regulator in tissue homeostasis and inflammation. However, the functions of iNOS on ISCs have not been clarified. Here, we aimed to investigate the expression pattern of inducible nitric oxide synthase (iNOS) within crypts and explore its function in the homeostatic maintenance of the ISC niche.

**Methods:**

Expression of iNOS was determined by tissue staining and qPCR. iNOS^−/−^ and Lgr5 transgenic mice were used to explore the influence of iNOS ablation on ISC proliferation and differentiation. Enteroids were cultured to study the effect of iNOS on ISCs in vitro. Ileum samples from wild-type and iNOS^−/−^ mice were collected for RNA-Seq to explore the molecular mechanisms by which iNOS regulates ISCs.

**Results:**

iNOS was physiologically expressed in Paneth cells. Knockout of iNOS led to apparent morphological changes in the intestine, including a decrease in the small intestine length and in the heights of both villi and crypts. Knockout of iNOS decreased the number of Ki67^+^ or BrdU^+^ proliferative cells in crypts. Loss of iNOS increased the number of Olfm4^+^ ISCs but inhibited the differentiation and migration of Lgr5^+^ ISCs in vivo. iNOS depletion also inhibited enteroid formation and the budding efficiency of crypts in vitro. Moreover, iNOS deficiency altered gluconeogenesis and the adaptive immune response in the ileum transcriptome.

**Conclusion:**

Paneth cell-derived iNOS is required to maintain a healthy ISC niche, and Knockout of iNOS hinders ISC function in mice. Therefore, iNOS represents a potential target for the development of new drugs and other therapeutic interventions for intestinal disorders.

**Supplementary Information:**

The online version contains supplementary material available at 10.1186/s12967-023-04744-w.

## Background

The most robustly proliferative and renewing tissue in mammals, the intestinal epithelium contains many intestinal stem cells (ISCs) and progenitor cells. The differentiation process of intestinal epithelial cells (IECs) depends on ISCs within intestinal crypts [1, 2]. ISCs are under precise regulation by their niche, which consists of Paneth cells, macrophages, myofibroblasts and other intestinal mesenchymal cells [3, 4].Among them, Paneth cells can provide ISCs with essential niche signals, such as epidermal growth factor (EGF), wingless-type MMTV integration site family member 3 (Wnt3), and Notch, to maintain ISC homeostasis [5]. Paneth cells regulate intestinal mucosal immunity by secreting antimicrobial peptides, including defensin and lysozyme, thus maintaining intestinal homeostasis by modulating the inflammatory response [6, 7]. Paneth cells can also directly dedifferentiate to replace the missing ISCs required for tissue repair or regulate the transformation and renewal of quiescent ISCs (QISCs) to active ISCs (AISCs) after radiation injury to promote the regeneration and repair of the intestinal epithelium [8, 9]. Therefore, Paneth cells are crucial for maintaining ISC homeostasis.

Nitric oxide (NO) is an important gas signaling molecule in vivo that has various biological functions, such as protecting the intestinal mucosa, dilating blood vessels, inhibiting platelet aggregation, killing tumor cells and regulating immunity [10]. However, excessive NO has cytotoxic effects [11]. There are three different types of NO synthases (NOSs) that can catalyze arginine to form NO in vivo, namely, inducible NOS (iNOS), which has the strongest catalytic ability to produce NO, endothelial NOS (eNOS) and neural NOS (nNOS) [12, 13]. Early studies have shown that iNOS is mainly expressed in macrophages and endothelial cells, but iNOS can also be expressed in epithelial cells of the gastrointestinal tract, kidney and other organs, as demonstrated in a mouse model of septic shock [14]. In mucosal ischemia‒reperfusion injury, iNOS levels in Lgr5^+^ ISCs are downregulated to protect ISC function [15]. However, the origin and effect of iNOS on the physiological maintenance of ISCs have not been well investigated.

In this study, we explored the expression pattern and effects of iNOS on the dynamic balance of epithelial renewal in the small intestine using iNOS^−/−^ mice and ISC-related transgenic mice. We found that iNOS was physiologically expressed in Paneth cells of the small intestine, and Knockout of iNOS significantly affected ISC activity in vivo and in vitro, indicating that iNOS was required for the homeostatic maintenance of the ISC niche under physiological conditions. These findings indicated that iNOS might be a target for the development of drugs for intestinal disorders.

## Methods

### Mice

*B6.129P2-Nos2*^*tm1Lau*^*/J* (iNOS^−/−^), *B6.129P2-Lgr5*^*tm1(cre/ERT2)*Cle^/*J* (Lgr5-EGFP-IRES-creERT2), and *B6;129S6-Gt(ROSA)26Sor*^*tm14(CAG−tdTomato)H*ze^/*J* (tdTomato) mice were purchased from The Jackson Laboratory (Bar Harbor, ME). The genotypes of these mice were determined by polymerase chain reaction (PCR) and DNA agarose gel electrophoresis. When the progeny of iNOS^−/−^, Lgr5-tdTomato and Lgr5-tdTomato; iNOS^−/−^ mice reached 6 to 8 weeks of age, male mice were selected for subsequent studies along with their wild-type littermate controls. All mice were maintained in a specific pathogen-free (SPF) facility with a 12 h light/dark cycle and were allowed standard food and water ad libitum. All experimental procedures were carried out in accordance with the guidelines for the Care and Use of Laboratory Animals and approved by the Ethics Committee of Sichuan Cancer Hospital & Institute (SCCHEC-04-2023-009).

### Tissue collection and immunostaining

Intestine samples were collected, fixed with 4% cold paraformaldehyde (BL539A, Biosharp, China) for 72 h, dehydrated and paraffin embedded using the standard histological protocol of our laboratory. Sections of 4 μm thickness were used for hematoxylin–eosin (H&E) (E607318, BBI, China), immunohistochemistry (IHC) (PV-6001/6002, ZSGB-BIO, China) and immunofluorescent (IF) staining. Antigen retrieval was performed using boiled TRIS–EDTA Antigen Retrieval Solution (BL618A, Biosharp) for 20 min, and tissues were blocked with phosphate buffered solution (PBS, SH30256.01, HyClone, USA) containing 1% bovine serum albumin (BSA, A7906, Sigma‒Aldrich, USA) and subsequently incubated with specific primary antibodies against Olfm4 (39141S, CST, USA), BrdU (ab152095, Abcam, UK), Ki67 (ab1667, Abcam), pHH3 (9718 T, CST), FABP1 (13368 T, CST), lysozyme (ab108508, Abcam), chromogranin A (60,135–1-1 g, Proteintech, USA), DCLK1 (21,699–1-AP, Proteintech), β-catenin (610,153, BD Biosciences, USA), and p27 Kip1 (3686 T, CST, USA) at a 1:200 dilution ratio according to the manufacturer’s instructions. Goblet cells were stained with an Alcian Blue and Nuclear Fast Red staining kit (E670107, BBI). Measurements for each quantitative outcome were collected from 30 crypts or villi per mouse analyzed and from more than 3 independent fields of the ileum.

### Organoid culture

Fresh intestinal tissues were flushed with ice-cold PBS and opened longitudinally on ice. Intestines were cut into 3 to 5 mm pieces, washed briefly with PBS, and placed into PBS supplemented with 5 mM EDTA (25,300,096, Invitrogen, USA). The tissue was incubated for 30 min at 4 °C and then washed with PBS. Villi and crypts were isolated by vigorous shaking and were passed through a 70-μm strainer to separate crypts. Individual crypts were collected by centrifugation at 4 °C and 800 ×*g* for 3 min. Crypts, mixed with Matrigel (354,230, Corning, USA), were seeded into 96-well flat-bottom plates (100 crypts/well). The plates were incubated at 37 °C for 10 min. Then, IntestiCult™ Organoid Growth Medium (06005, STEMCELL Technologies, Canada) supplemented with 100 μg/mL streptomycin and 100 units/mL penicillin (15,140-122, Invitrogen) was added to the wells. 1400W (HY-18731, MCE, China), the specific inhibitor of iNOS, was used to treat intestinal organoids in order to mimic the deficiency of iNOS.

### Image capture and data collection

Images of H&E and IHC staining on slides and of organoids in culture plates were captured by M5000 (Thermo Fisher, USA), Cytation 5 (BioTek, USA) or BX53 (Olympus, Japan) microscope. Image parameters were generated by ImageJ (NIH, USA) for each image. IF staining images were captured by a A1R confocal microscope (Nikon, Japan). Each value was calculated based on at least three independent replicates.

### Quantitative polymerase chain reaction

Total RNA extraction was performed using RNAiso Plus (9109, TaKaRa, Japan) following the manufacturer’s recommendations, and then the total RNA concentration was determined using a NanoDrop2000 spectrophotometer (Thermo Scientific). Hifair II 1st Strand cDNA Synthesis SuperMix (11137ES60, YEASEN, China) was used to synthesize cDNA, and qPCR was performed using Hieff qPCR SYBR Green Master Mix (11203ES08, YEASEN). The expression levels of *Nos2, Lgr5, MKi67, Lyz2, Wnt3a*, etc., were measured by qPCR using a C1000 instrument (Bio-Rad, USA). Primer sequences used in this study were listed in Table 1. Gene expression results were normalized to that of β-actin, and relative expression was determined by the 2^−ΔΔCt^ method.

**Table 1:** Primer sequences used for qPCR examination

Gene name	Forward primer	Reverse primer
Actb	CTTCTTTGCAGCTCCTTCGTT	TTCTGACCCATTCCCACCA
Nos2	CACAGAGGGCTCAAAGGAGG	AAAGTGGTAGCCACATCCCG
Lgr5	CAGGTCAATACCGGAGCGAG	GCGAGGCACCATTCAAAGTC
MKi67	ATCATTGACCGCTCCTTTAGGT	GCTCGCCTTGATGGTTCCT
Lyz2	ATGGAATGGCTGGCTACTATGG	ACCAGTATCGGCTATTGATCTGA
Wnt3a	TGGAACTGTACCACCATAGATGAC	ACACCAGCCGAGGCGATG
Cela2a	TACCCCACTTATGAGGTGGAG	GTCTGATAGTTGCTGAGGCAAT
Prss2	TATCAGGTGTCCCTAAATGCTGG	GGATGCGGTATTTGTAGCAGT
Try5	GAATTCACTCCTGTTCCTGGC	CAGAGACACCTGGTAGGGGA
Cel	CGCCTGGAGGTTCTATTTCTTG	GCCCTTGAAGATGTCAACAGA
S100g	ATGTGTGCTGAGAAGTCTCCT	CGCCATTCTTATCCAGCTCCTT
Krcap3-1	CTGCCCACATGAGATCAGCC	GGCAAGAGTTGAGCAGCCA
Bco1	ATGGGGAGGTCTTCTACAGGA	GATGGTGTGAGACAAGTAGGAGA
Dll4	TTCCAGGCAACCTTCTCCGA	ACTGCCGCTATTCTTGTCCC
H2-Aa	TCAGTCGCAGACGGTGTTTAT	GGGGGCTGGAATCTCAGGT
H2-DMb1	ACCCCACAGGACTTCACATAC	GGATACAGCACCCCAAATTCA
H2-Ab1	AGCCCCATCACTGTGGAGT	GATGCCGCTCAACATCTTGC
Nod2	CAGGTCTCCGAGAGGGTACTG	GCTACGGATGAGCCAAATGAAG
Pck1	CTGCATAACGGTCTGGACTTC	CAGCAACTGCCCGTACTCC
G6pc1	CGACTCGCTATCTCCAAGTGA	GTTGAACCAGTCTCCGACCA
Atf3	GAGGATTTTGCTAACCTGACACC	TTGACGGTAACTGACTCCAGC
Fbp1	CACCGCGATCAAAGCCATCT	AGGTAGCGTAGGACGACTTCA

### RNA-Seq assay

Ileum tissue samples (from three wild-type mice and three iNOS^−/−^ mouse littermates) were isolated, gently washed with DEPC-treated water (10601ES76, YEASEN), frozen and then used for transcriptome RNA-Seq. Total RNA extraction, RNA integrity evaluation, library construction, and sequencing were performed according to the manufacturer’s standard protocol. RNA-seq and analysis were conducted by OE Biotech Co., Ltd. (Shanghai, China). Differentially expressed genes (DEGs) were identified using the absolute value of log2 (ratio) ≥ 1 as the threshold. The t test threshold (*P* values < 0.05) and fold-change threshold (> 1.5 or < 0.5) were set as the thresholds for significantly DEGs. Gene Ontology (GO) enrichment and Kyoto Encyclopedia of Genes and Genomes (KEGG) pathway database analyses were performed. MetaboAnalyst of DEGs was performed using R based on the hypergeometric distribution. Gene set enrichment analysis (GSEA) was performed to determine pathways that were significantly enriched in DEGs for each group compared with those in the GSEA molecular signature database. RNA-Seq results were further validated by qPCR.

### Statistical analysis

All statistical analyses were performed using GraphPad Prism 9 (GraphPad Software, USA). The results are expressed as the mean ± standard deviation (SD) values. Statistical significance was determined using the *Student’s t* test for two-group comparisons and one-way ANOVA for three or more groups. P values of less than 0.05 were considered statistically significant.

## Results

### iNOS was physiologically expressed in Paneth cells

**Fig. 1:**
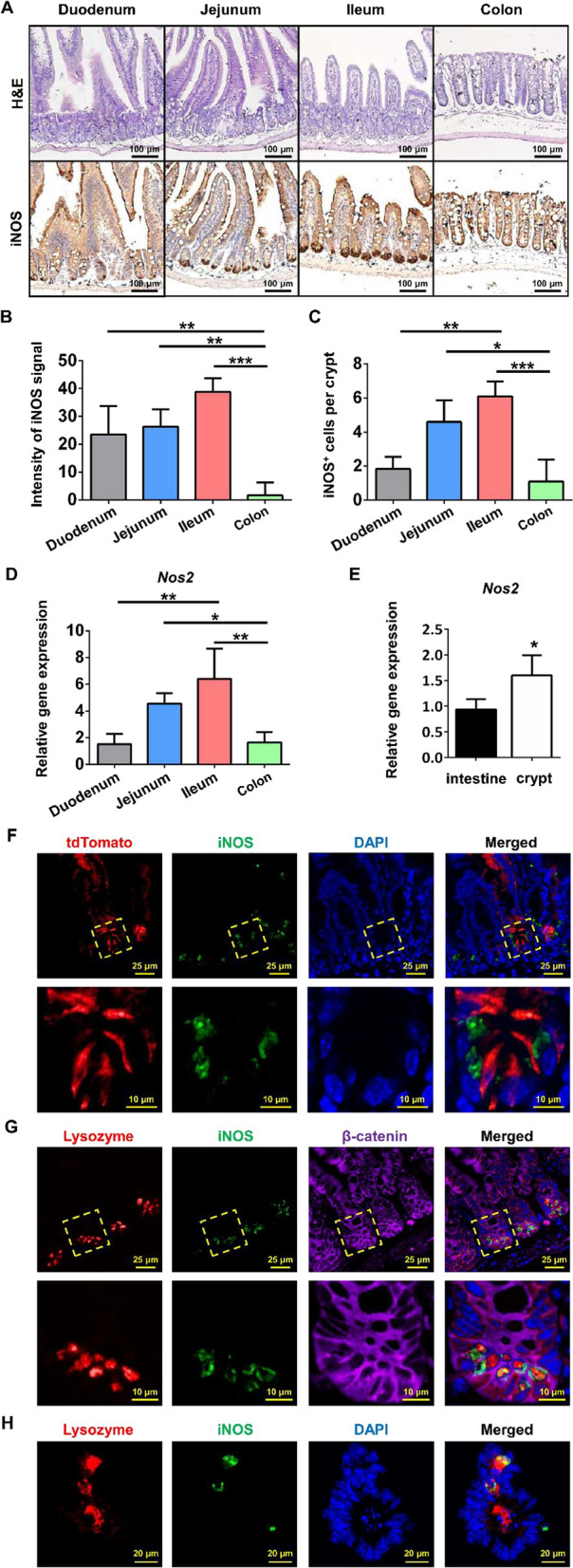
Distribution and localization of iNOS in the small intestine of mice. **A** Representative IHC staining images of iNOS in different intestinal segments from wild-type mice. Bar = 100 μm. **B**, **C** Quantification of the staining intensity of iNOS and the number of iNOS positive cells in different segments. *P < 0.05, **P < 0.01, ***P < 0.001. **D** qPCR analysis of iNOS mRNA levels in different intestinal segments. The ileum had the highest level of iNOS mRNA, and the differences in iNOS expression between the duodenum vs. Jejunum, Duodenum vs. Colon and Jejunum vs. Ileum were not significant (*P* > 0.05). *P < 0.05, **P < 0.01, n = 3. **E** qPCR analysis of iNOS mRNA expression in the whole small intestine and crypts. **F** Lgr5-tdTomato (red), iNOS (green) and DAPI (blue) positive signals at the cryptal base of Lgr5-tdTomato mice. **G** Lysozyme granules (red), iNOS (green) and β-catenin (purple) positive signals at the crypt base of wild-type mice. **H** Cultured wild-type enteroid was stained by lysozyme (red), iNOS (green) and DAPI (blue) at day 4. Bar = 20 μm

First, we examined the expression pattern of iNOS in different intestinal segments by IHC staining and qPCR analysis. The highest protein and mRNA expression level of iNOS in small intestines was found in the ileum, followed by the jejunum, and the lowest expression level was found in the duodenum and colon (Fig. 1A–D). To determine iNOS mRNA levels in different parts of the intestinal epithelium, we isolated and purified individual intestinal crypts, and qPCR data showed that iNOS mRNA was mainly expressed in the crypts of small intestine (Fig. 1E). Moreover, IF staining in Lgr5-tdTomato mice was performed to confirm the histological localization of iNOS. iNOS was distributed outside of the tdTomato^+^ Lgr5^−^positive AISCs, and no colocalization of iNOS of tdTomato was observed (Fig. 1F). Furthermore, subcellular localization analysis of iNOS in intestine and enteroids showed that iNOS positive signals were mainly distributed in the space between the nucleus and lysozyme granules, while some iNOS positive signals colocalized with lysozyme particles in vivo and in vitro (Fig. 1G,H).

### Ablation of iNOS impaired the morphological characteristics of the small intestine

**Fig. 2:**
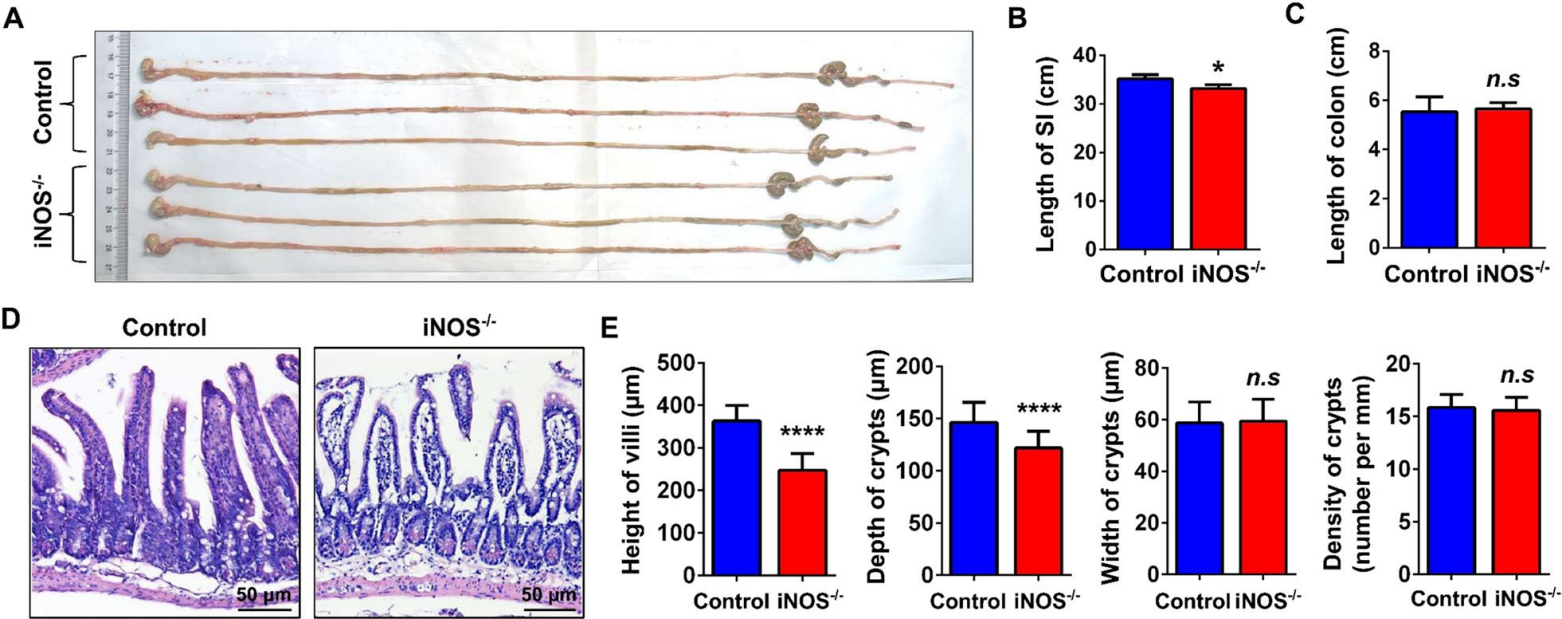
iNOS deletion impaired the morphology of the intestine. **A** Gross images of gastrointestinal tract samples from the control and iNOS^−/−^ groups. **B** The length of the small intestine (SI) of iNOS^−/−^ group mice was significantly shorter than that of control group mice (n = 3). **C** There was no difference in the length of colons from the control and iNOS^−/−^ groups (n = 3). **D** Representative images of hematoxylin and eosin staining in the ileum for the control group and iNOS^−/−^ mice. Bar = 50 μm. **E** Statistical analysis of villus height, crypt depth, crypt width and crypt density between the control group and iNOS^−/−^ group. *n.s*: *P* > 0.05, *P < 0.05, ****P < 0.0001, n = 3

To explore the role of iNOS in the intestinal epithelium, iNOS^−/−^ mice were used and genotyped for subsequent experiments (Additional file 1: Fig. S1). Decreased mRNA level of iNOS was confirmed by qPCR with crypts (Additional file 1: Fig. S2). We randomly selected wild-type mice (control group) and iNOS^−/−^ mice (iNOS^−/−^ group) littermates and collected gastrointestinal tracts of mice. Gross images demonstrated that iNOS^−/−^ mice had a shorter GI tract than control mice (Fig. 2A). iNOS^−/−^ mice had significantly shorter small intestines than controls but the same colonic length (Fig. 2B, C). H&E staining also showed that both the height of villi and the depth of crypts were decreased in NOS^−/−^ mice compared with control mice. However, we observed no differences in crypt density (Fig. 2D, E). These results suggested that iNOS was involved in the maintenance of homeostasis and normal tissue structure in the small intestinal epithelium.

### Knockout of iNOS decreased the quantity of proliferative cryptal cells

**Fig. 3:**
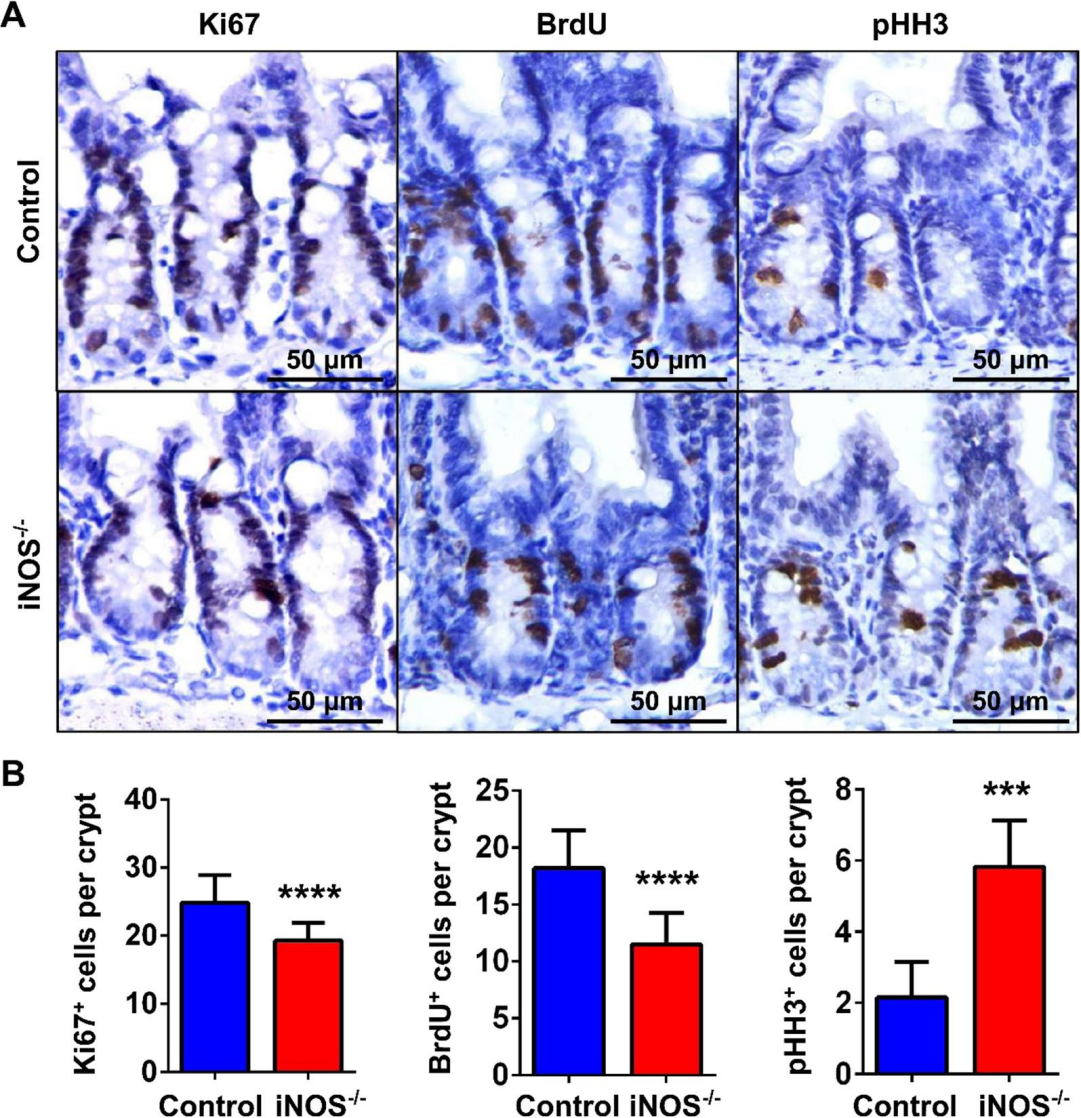
Knockout of iNOS changed the proliferative status within intestinal crypts. **A** Representative images of immunohistochemical staining for Ki67, BrdU and pHH3 in the crypts of the mouse ileum. Tissues were collected from the control group and iNOS^−/−^ group mice at 1.5 h after BrdU injection. Bar = 50 μm. **B** Quantification of cells positive for Ki67, BrdU, and pHH3 staining and statistical analysis between the control and iNOS^−/−^ groups. ***P < 0.001, ****P < 0.0001, n = 5

To identify the function of iNOS in the homeostasis of intestinal epithelial renewal, we first analyzed its effect on proliferative cells. IHC staining showed that iNOS^−/−^ mice had fewer Ki67^+^ and BrdU^+^ actively proliferating cells, indicating that these mice had fewer cells in mitotic interphase, especially S-phase, than normal mice. Nevertheless, iNOS^−/−^ mice had more pHH3^+^ cells that were in late G2 or M-phase of the cell cycle than the control group (Fig. 3). In addition, we also found a decrease of p27 Kip1 (Additional file 1: Fig. S3) in iNOS^−/−^ mice. These data suggested that iNOS knockout leads to an abnormal proliferation in cryptal epithelial cells and inhibits crypt cell proliferation by hindering the G2/M transition in mice.

### Knockout of iNOS inhibited the proliferation and differentiation of ISCs

**Fig. 4:**
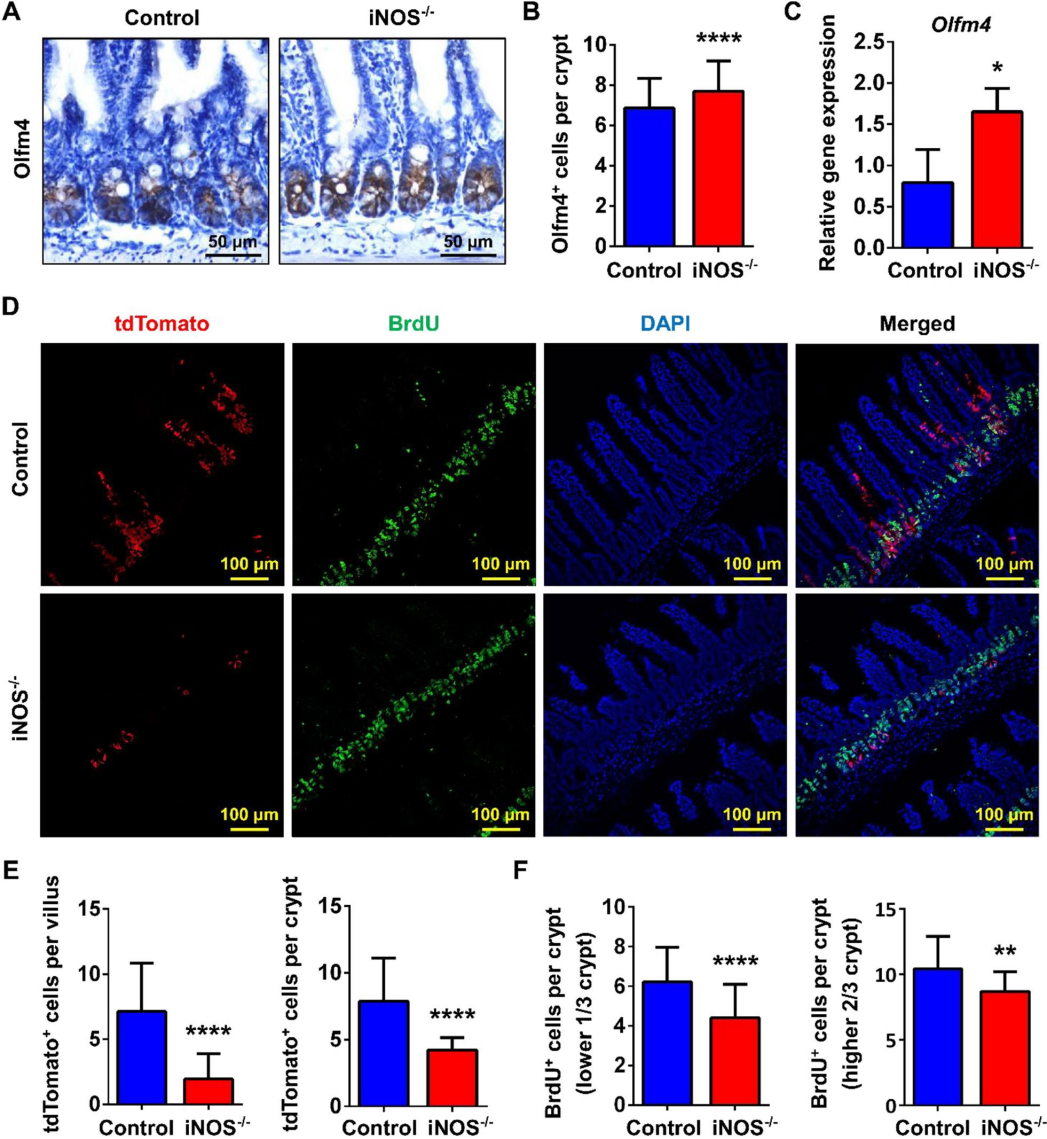
iNOS deletion inhibited the quantity and differentiation of AISCs in the intestinal epithelium. **A** Immunohistochemistry staining of Olfm4 in ileum tissues from the control group and iNOS^−/−^ group. Bar = 50 μm. **B** Statistical analysis of Olfm4-positive cells between the two groups in Fig. 4A. **C** qPCR analysis of the mRNA level of Olfm4 gene in ileum samples. *: *P* < 0.05.** D** Confocal images of tdTomato (red), BrdU (green) and DAPI (blue) positive signals in Lgr5-tdTomato and Lgr5-tdTomato; iNOS^−/−^ littermate mice. Bar = 100 μm. **E** Comparison of tdTomato-positive cells in villus or crypts in the control and iNOS^−/−^ groups. **F** Respective analysis of BrdU^+^ cells in the lower 1/3 or the upper 2/3 of crypts. *n.s*: *P* > 0.05, **P < 0.01, ****P < 0.0001, n = 3

To further study how iNOS deletion might impair ISC function, we assessed the expression of AISC markers, including Olfm4 and Lgr5. Contrary to expectations, both immunohistochemistry and qPCR results showed that the active ISC marker “Olfm4” was upregulated in the crypts of iNOS^−/−^ mice (Fig. 4A–C). Such an increase of Olfm4^+^ cells might be considered compensatory due to a reduction of Lgr5^+^ ISCs. Lineage-tracing assays demonstrated that the migration of tdTomato^+^ Lgr5^+^-derived progenitor cells or daughter cells was obstructed in iNOS^−/−^ mice compared with control mice (Fig. 4D, E). Indeed, BrdU^+^ cells were decreased in both the upper 2/3 and the base of the crypt in iNOS^−/−^ mice (Fig. 4F). This pattern might explain the shortened small intestinal villus height and crypt depth in iNOS^−/−^ mice. The results suggested that ablation of iNOS led to the decreased number of Lgr5^+^ ISC-derived cells and inhibited their proliferation capacity.

**Fig. 5:**
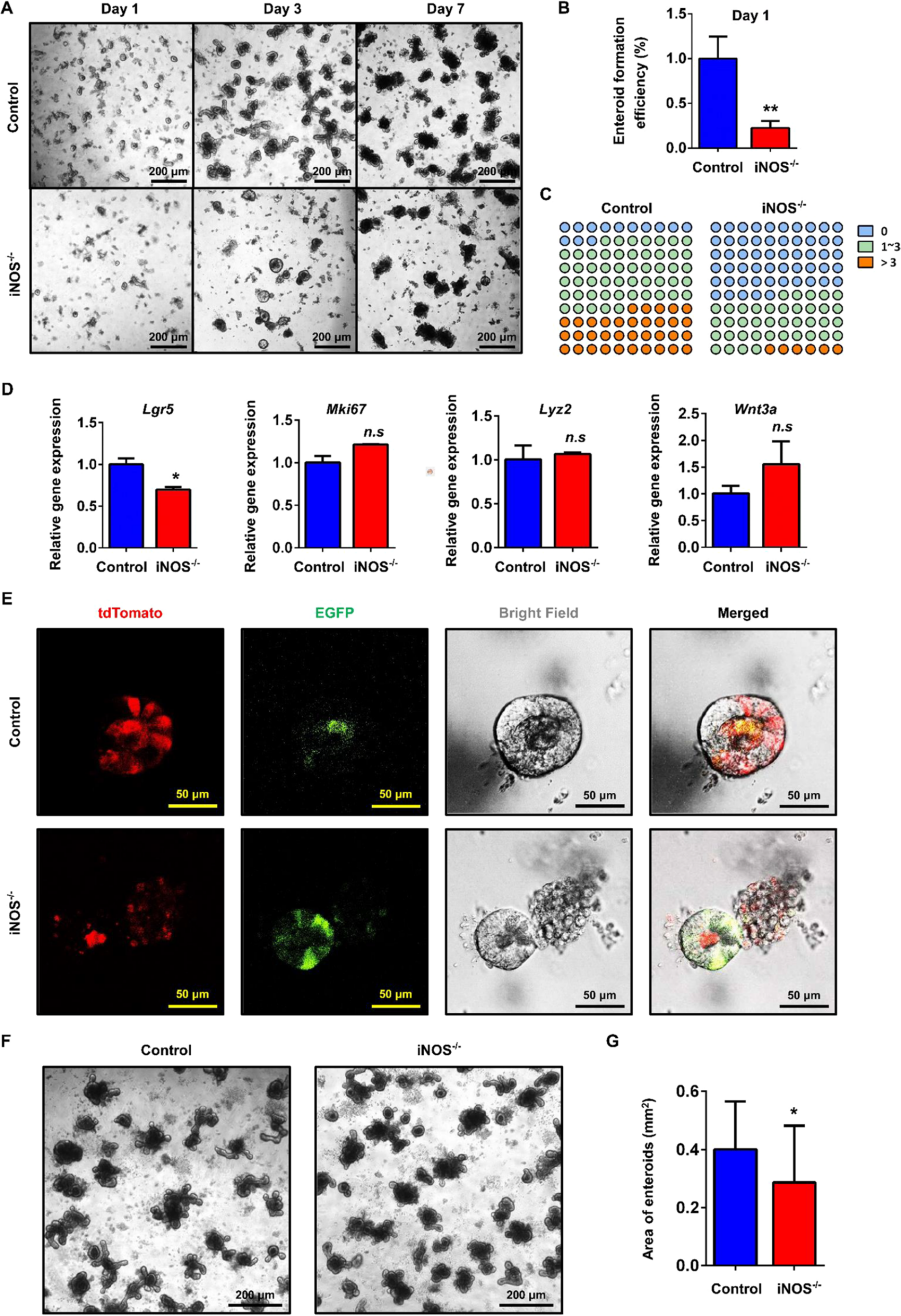
iNOS deficiency inhibited the growth of enteroids. **A** Representative results for the growth of enteroids derived from wild-type and iNOS^−/−^ mice on days 1, 3, and 7 after seeding. Bar = 200 μm. **B** The enteroid formation efficiency of the iNOS^−/−^ group was clearly decreased compared to that of the control group at 24 h after culture. **C** The budding numbers of enteroids between the control group and iNOS^−/−^ group were divided into 0, 1–3 or > 3 per organoid. iNOS^−/−^ group had less budding capacity. **D** RNA samples from the enteroids of the two groups at Day 4 were used for qPCR analysis of *Lgr5*, *MKi67*, *Lyz2* and *Wnt3a*. *n.s*: *P* > 0.05, *P < 0.05, n = 3. **E** Enteroids derived from the control and iNOS^−/−^ groups in Lgr5-tdTomato mice on day 1 after seeding. Bar = 50 μm. **F** Cultured enteroids were treated with 1400W, and the growth was significantly inhibited. Bar = 200 μm. **G** Areas of enteroids in the two groups. *P < 0.05

To further validate the importance of iNOS in maintaining the homeostasis of ISC niche, we cultured enteroids using crypts derived from wild-type mice and iNOS^−/−^ mice or Lgr5-tdTomato; iNOS^−/−^ mice ex vivo. We found that wild-type mouse-derived crypts could form enteroids at day 1 after seeding and that these enteroids budded at day 3. However, crypts from iNOS^−/−^ mice were less capable of forming enteroids and had fewer crypt-like buds at the same timepoints (Fig. 5A–C). At day 4, we digested the Matrigel and collected enteroids for qPCR analysis. The results showed that Lgr5 mRNA was significantly decreased in the iNOS^−/−^ group, while the expression of Mki67, Lyz2 and Wnt3a exhibited a tendency toward upregulation (Fig. 5D). In addition, there were tdTomato-labeled Lgr5-tdTomato positive ISCs and their progenies in enteroids derived from Lgr5-tdTomato mice at day 1 after seeding. However, there were only some EGFP-positive ISCs in those enteroids derived from Lgr5-tdTomato; iNOS^−/−^ mice, indicating a decreased differentiation capacity of Lgr5^+^ ISCs under the loss of iNOS within Paneth cells (Fig. 5E). Treatment with 1400W, the specific iNOS inhibitor, also blocked the growth of enteroids (Fig. 5F, G). This result was consistent with that of the organoid formation assay. Taken together, these results indicated that iNOS signals were essential for the maintenance of Lgr5^+^ ISC function.

### Knockout of iNOS impaired the differentiation of ISCs

**Fig. 6:**
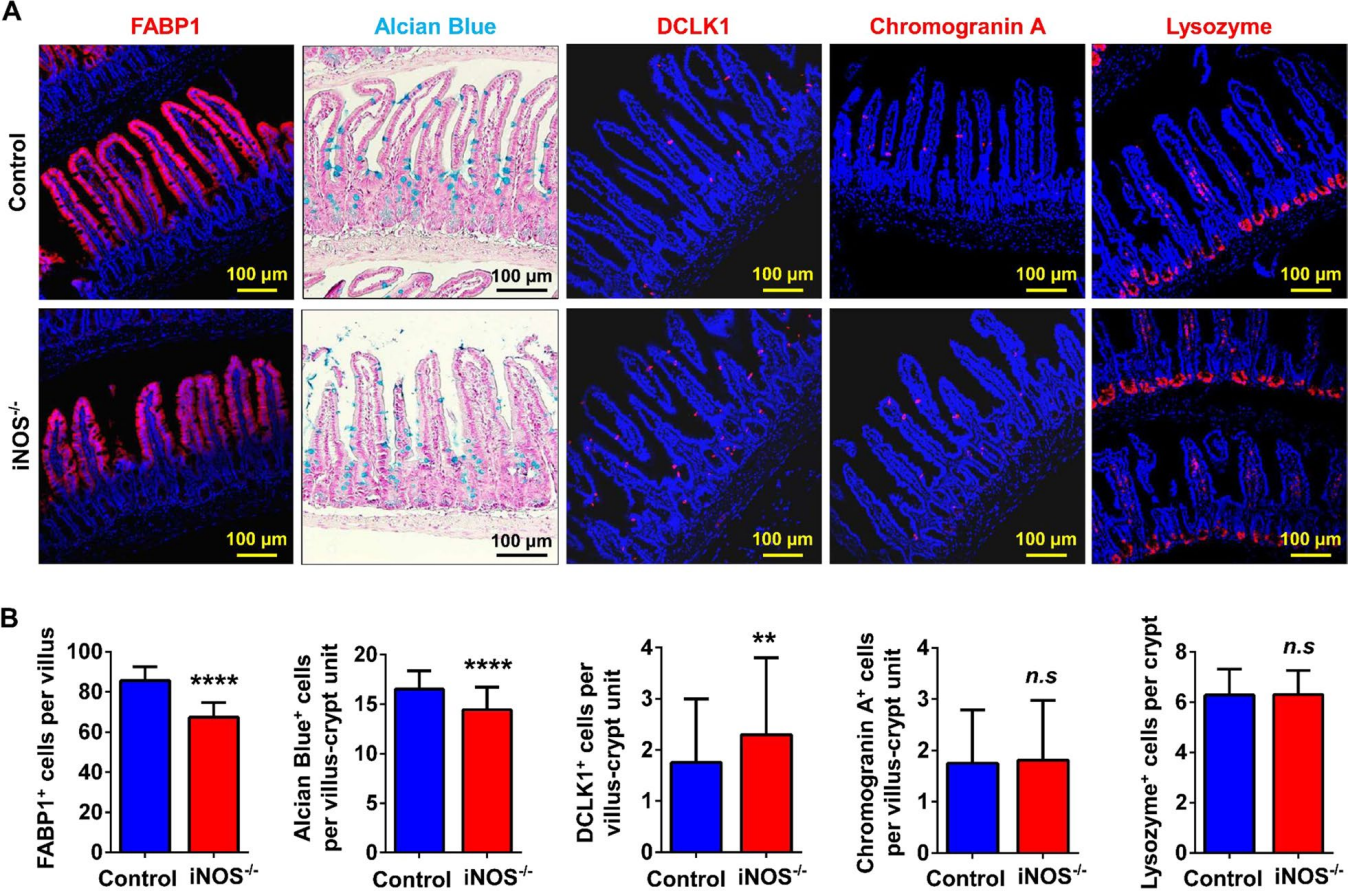
Knockout of iNOS affected the differentiation pattern in the intestinal epithelium. **A** Staining of absorptive epithelial cells (FABP1), goblet cells (Alcian blue), Tuft cells (DCLK1), enteroendocrine cells (Chromogranin A) and Paneth cells (lysozyme) between the control group and iNOS^−/−^ group. Bar = 100 μm. **B** Statistical analysis of different subpopulations of IECs in Fig. 6A. *n.s*: *P* > 0.05, **P < 0.01, ****P < 0.0001, n = 5

To determine the influences of iNOS deletion on the subpopulations of IECs, we analyzed different lineage markers of IECs and found a significant decrease of FABP1^+^ absorptive epithelial cells and Alcian blue-positive goblet cells in iNOS^−/−^ mice, whereas DCLK1^+^ Tuft cells were increased compared to control mice. However, the numbers of Chromogranin A^+^ enteroendocrine cells and lysozyme positive Paneth cells did not change significantly (Fig. 6). In addition, qPCR result showed that the mRNA level of Dll4 was decreased (Additional file 1: Fig. S5H), suggesting an inhibition of Notch pathway caused by iNOS deficiency. These results indicated that iNOS might be required for the differentiation of ISCs into absorptive epithelial cells and goblet cells.

### Knockout of iNOS altered gluconeogenesis and the immune response in the ileum

**Fig. 7:**
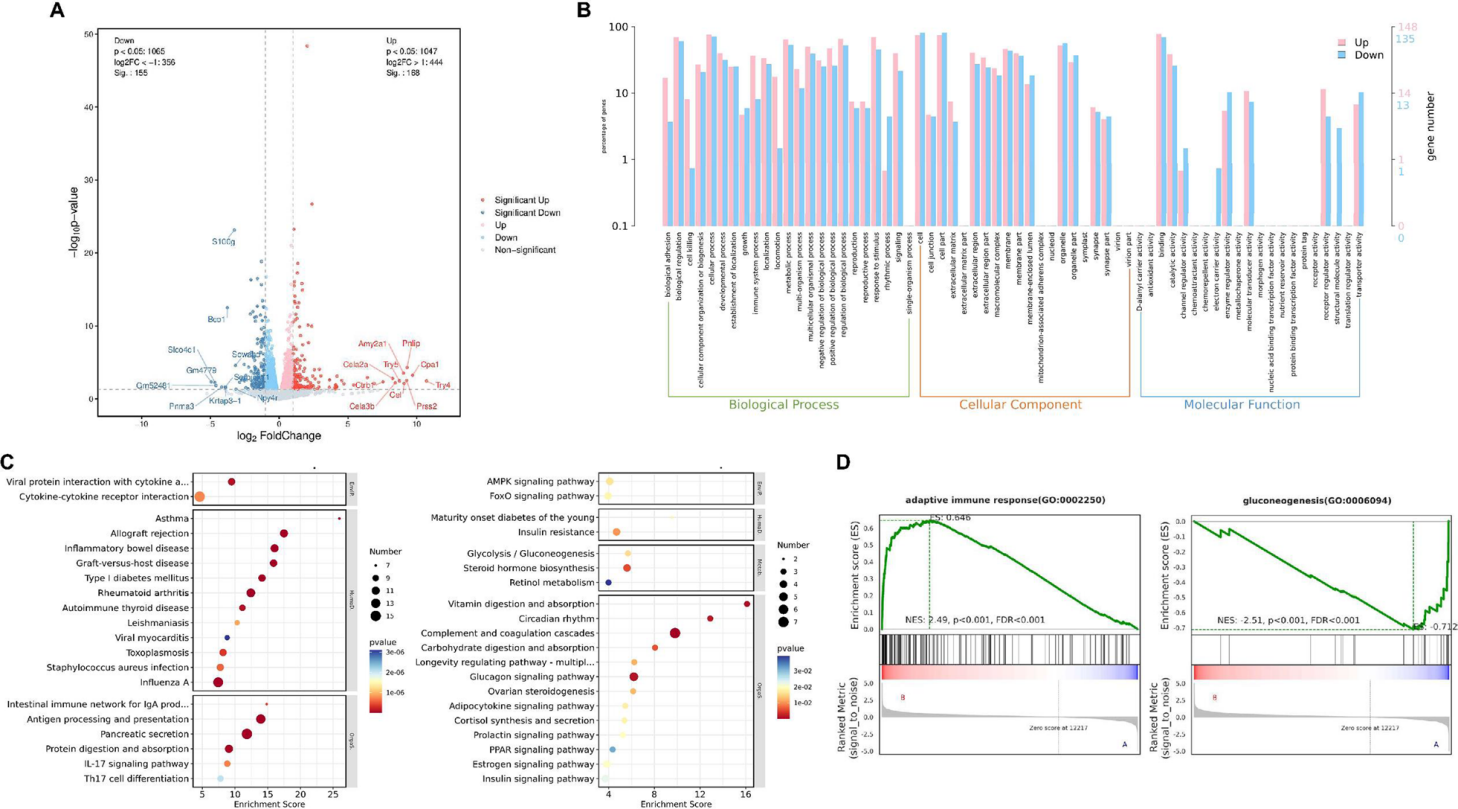
iNOS deficiency altered gluconeogenesis and the adaptive immune response in the ileum transcriptome. **A** Volcano plot depicting transcriptomics data with dotted line marking *P* = 0.05 on y-axis and fold change of greater than 1 on X-axis. The top ten gene names are shown (red: upregulated, blue: downregulated). **B** Gene Ontology analyses of RNA-seq data showed significant changes in pathways in the ileum from the control and iNOS^−/−^ groups. **C** Kyoto Encyclopedia of Genes and Genomes enrichment analysis (upregulated and downregulated differential) bubble plot. The X-axis is the enrichment score, and the y-axis is the pathway information of the top 20. The larger the bubble, the greater the number of differential proteins contained in the entry. When the color of bubbles changes from red to yellow and blue, the p value decreases. **D** GSEA of genes upregulated or downregulated in association with iNOS depletion

To further elucidate the influences of iNOS on the molecular pathways of the ISC niche, we performed mRNA sequencing using ileums from the control and iNOS^−/−^ groups. We found that 168 genes were upregulated and 155 genes were downregulated in the iNOS^−/−^ group compared with the control group (Additional file 1: Fig. S4). To explore DEGs, we constructed a volcano map and marked the top 10 up- or downregulated differentially expressed proteins (Fig. 7A). The abscissa of the volcano map was log2 (FoldChange), while the ordinate was -log10 (*P* value). The red dots represented upregulated DEGs, the blue dots showed downregulated DEGs, and the gray dots meant DEGs without significant differences. Parts of DEGs were validated by qPCR examination (Additional file 1: Fig. S5A–G).

To investigate the biological functions of iNOS, GO enrichment analysis was performed including terms for biological processes, cellular components, and molecular functions (Fig. 7B). We found that iNOS deletion was involved in GO level 2 terms, such as biological regulation, cellular process, metabolic process and response to stimulus in biological processes. The most significantly enriched cellular component category terms were cell and cell part, while binding and catalytic activity were highlighted in the molecular function category. KEGG pathway enrichment showed that upregulated genes were mainly enriched in allograft rejection and in antigen processing and presentation. Among downregulated DEGs, we found that vitamin digestion and absorption, glucagon signaling pathway, complement and coagulation cascades were more closely associated with the ablation of iNOS (Fig. 7C, Additional file 1: Fig. S5I–L). Inflammatory factors were also changed in iNOS knockout mice (Additional file 1: Fig. S6).

Furthermore, GSEA enrichment analysis showed that genes differentially expressed in these two groups were significantly enriched in pathways associated with the adaptive immune response (upregulated) and gluconeogenesis (downregulated), as shown in Fig. 7D and Additional file 1: Fig. S5M–P. Although the absolute value of the normalized enrichment scores of downregulated genes in the glycolytic process were lower than those of genes in the other two terms, the enrichment of downregulated genes in this process was still relevant in iNOS^−/−^ mice (Additional file 1: Fig. S7).

## Discussion

ISCs play a crucial role in the renewal and regeneration of the intestinal epithelium. There are at least two subpopulations of ISCs, including Lgr5-marked AISCs and Bmi1-, mTert-, Hopx-, and Lrig1-marked QISCs, which are in the “ + 4” position of crypts [16–21]. AISCs, also called cryptal basal columnar cells (CBCs), are responsible for the renewal of the intestinal epithelium under physiological conditions, while QISCs rapidly proliferate and differentiate to supplement the AISC pool after injury, thus restoring the integrity of the intestinal epithelium [20, 21]. However, many underlying regulatory mechanisms of the ISC niche remain unclarified. In this study, we reported that iNOS was indispensable for the normal function of ISCs in physiological circumstances. We found that iNOS was primarily expressed in Paneth cells, especially within the mouse ileum. Conversely, iNOS deletion led to inhibited ISC proliferation and the migration and differentiation towards absorptive epithelial cells and goblet cells, which resulted in abnormal morphological structures of the small intestine. In addition, loss of iNOS hindered enteroid formation and budding in vitro. These results confirmed that an appropriate level of iNOS expression was required in Paneth cells for the maintenance, normal self-renewal, and differentiation of ISCs under physiological conditions.

iNOS, namely, NOS2, is a small molecule that exhibits wide roles in various cellular and molecular responses through a cGMP-mediated signal transduction pathway. Reportedly, iNOS is induced by endotoxins and cytokines, which function in synergy with bacterial lipopolysaccharides (LPS), tumor necrosis factor (TNF) and interleukin 1β (IL1β). Previously, it has also been shown that iNOS is barely expressed under physiological conditions, and its expression can be rapidly induced to modulate vascular endothelial cells, myocardial cells, vascular smooth muscle cells, and neurons in response to inflammation and injury. For example, Paneth cells were identified as one of the major iNOS-expressing cells in TNFa or LPS challenged shock models and during Salmonella infection [14]. However, until now, there has been no study about whether iNOS affects Paneth cells and ISCs in intestinal homeostasis and how these effects may occur.

Paneth cells are one of the most important cells composing the ISC niche. They were first discovered by Gustav Schwalbe in 1872 [22], and then Joseph Paneth [23] described their characteristics and renamed them “Paneth cells” in 1888. In 1969, while studying ISCs and their progenitor cells, Hazel Cheng et al. [24] found that Paneth cells are renewed in the mouse small intestine. Paneth cells were reported to constitute the niche of Lgr5^+^ ISCs for the first time in 2011 [5]. In this study, we demonstrated that iNOS was physiologically expressed in Paneth cells and localized between the nucleus and lysozyme granules. Additionally, we further confirmed that iNOS deficiency had negative influences on the homeostasis of the intestinal epithelium by hindering ISC function, which might contribute to further elucidation of the anatomy and the regulatory mechanisms within the ISC niche.

Interestingly, we found that there were increased Olfm4^+^ ISCs, which was not consistent with our hypothesis (Fig. 4A–C). Olfm4 is a glycoprotein belonging to the olfactomedin family that is highly expressed in the intestine and used as a marker of ISCs [25, 26]. However, the spectrum of cells in which Olfm4^+^ is expressed is larger than that of Lgr5^+^ CBCs; thus, Lgr5 is now preferred for the identification of AISCs [16, 27, 28]. Since Lgr5^+^ ISCs are intermingled between Paneth cells, we postulate that they are more easily affected by the ablation of iNOS within Paneth cells. Both lineage tracing and qPCR results finally support this hypothesis (Figs. 4D, 5E). Organoids are a powerful model in which to study the interaction between different cell subpopulations. Because iNOS is also expressed by macrophages in the lamina propria of the intestine, the results of in vivo studies might be affected by mesenchymal iNOS signals [29, 30]. Therefore, we cultured enteroids from the ileums of wild-type and iNOS^−/−^ mice. This allowed us to confirm the necessity of iNOS in the homeostatic maintenance of the ISC niche, and poorer enteroid growth process was observed in iNOS^−/−^ mice (Fig. 5A). In addition, the administration of 1400W (an iNOS inhibitor) also caused an apparent inhibition for the growth of enteroids (Fig. 5F, G).

The RNA-Seq and GSEA results showed that the upregulated DEGs were enriched in the adaptive immune response. This finding was consistent with the knowledge that iNOS often functions as an important inflammatory regulator. Moreover, Knockout of iNOS was closely related to the downregulated genes in the gluconeogenesis pathway. Gluconeogenesis is a metabolic pathway for the biosynthesis of new glucose or 6-phosphoglucose from nonsugar compounds when there is a deficiency of hexose in ISC niches [31]. Like liver and kidney, the intestine is also able to carry out gluconeogenesis and release glucose into the blood in a process called intestinal gluconeogenesis (IGN). Genetically activating IGN via overexpression of intestinal glucose-6-phosphatase can counteract the pro-obesity and pro-diabetes effects of consuming high-calorie foods [32]. Conversely, downregulation of IGN could limit the production of glucose metabolism substrates, resulting in dysfunction of target cells, including Paneth cells and ISCs. Paneth cell glycolysis is thought to provide lactate for mitochondrial oxidative phosphorylation in ISCs, thus supporting ISC function [33, 34]. GSEA also showed that downregulated genes were enriched in glycolytic processes in iNOS^−/−^ mice. Therefore, the Knockout of iNOS might affect ISC function via the interruption of glycolysis within Paneth cells. Once the level of iNOS is low, Paneth cell glycolysis level would be restricted and lactate production would be decreased accordingly, subsequently limiting the oxidative phosphorylation process of ISCs, causing abnormal ISCs function. L-arginine serves as the substrate of arginase 1 (Arg-1) and inducible nitric oxide synthase, which is also a semi-essential amino acid needed for cell proliferation and function in mammals [35]. Exogenous L-arginine supplementation has been reported to protect and promote ISCs function in intestinal injuries [36, 37], which might be caused by the enhanced activity of iNOS within Paneth cells.

There are still several shortcomings that need to be improved upon in future studies. First, we investigated the function of iNOS in intestinal crypts by global knockout iNOS in mice rather than Paneth cell-specific iNOS knockout, so we cannot rule out the influence of inflammatory mechanisms, cardiovascular system effects, and neurological effects related to iNOS knockout. However, the unavailability of a Loxp-flanked iNOS conditional knockout mouse line at present limits the specific reduction of iNOS expression in Paneth cells. Second, while we investigated the intrinsic iNOS contribution to maintenance of ISC niches and used iNOS inhibitor 1400W to confirm the requirement of iNOS for enteroid growth, the agonists of iNOS, for example, sodium nitroprusside (SNP) could also be used to modulate iNOS activity and confirm their roles in ISC regulation. Third, more molecular experiments could be conducted to verify and probe RNA-Seq-related data to provide more solid support for the influence of iNOS knockout on intestinal metabolism.

## Conclusion

In summary, the present study demonstrates for the first time that iNOS is physiologically expressed in the mouse ileum and is localized in the cytoplasm between the nucleus and lysozyme granules of Paneth cells. Knockout of iNOS changes the histology of the small intestine and hinders the proliferation and differentiation of AISCs in vivo and in vitro, and dysfunction of the biological processes gluconeogenesis and glycolysis was identified as a potential mechanism of these changes. Therefore, the current study proves that iNOS is required for the homeostasis of the ISC niche, indicating that iNOS is a new target for the development of drugs for intestinal disorders.

### Supplementary Information

**Additional file 1:** **Figure S1.** Phenotype characterization of iNOS^−/−^ mice. **A** Genotyping of iNOS^−/−^ mice was achieved by 1.5% agarose gel electrophoresis. Het = heterozygote, KO = knockout, NTC = no template control. **B** qPCR analysis of iNOS mRNA levels in wild-type mice and iNOS^−/−^ mice. **C** Comparison of wild-type and iNOS^−/−^ littermate body weights. *P < 0.05, n = 5. **Figure S2.** qPCR analysis of *Nos2* in WT and iNOS^−/−^ enteroids. Enteroids derived from the two groups were released and collected on day 4 after culturing, and then total RNA was extracted and used for qPCR testing. **P < 0.01. **Figure S3.** Loss of iNOS significantly decreased the protein level of p27 Kip1 in the intestinal crypts. Bar = 100 μm. **Figure S4.** Histogram of differentially expressed genes (DEGs) between the control group and iNOS^−/−^ group. There were 168 upregulated genes (pink) and 155 downregulated genes (blue) in the iNOS^−/−^ group compared with the control group. N = 3 for each group. **Figure S5.** qPCR validation for those genes implicated by RNA-Seq. **A**–**D** qPCR validation of the upregulated genes in RNA-Seq results. **E**–**G** qPCR validation of the genes that were downregulated in RNA-Seq. **H** qPCR results of the mRNA level of Dll4 gene, which was a component of Notch signaling pathway. **I**–**L** qPCR examination of adaptive immunity related genes. **M**–**P** qPCR validation of gluconeogenesis related genes. (*P < 0.05, ***P < 0.001). **Figure S6.** RNA-Seq data of inflammatory factors. The analysis of RNA-Seq data showed that mRNA levels of Tnf and Ifgn were significantly increased due to iNOS deficiency, but Il1b and Nfkb1 did not change significantly. (*P < 0.05). **Figure S7.** GSEA of genes downregulated in association with iNOS depletion. Differentially downregulated genes in the iNOS^−/−^ group were also closely related to glycolytic processes in addition to gluconeogenesis (n = 3).

## Data Availability

All summary level data supporting the findings of this study are available within the paper and its Additional information. Upon reasonable request, the corresponding author will provide access to any data used or analyzed during this study**.**

## Abbreviations

iNOS: Inducible nitric oxide synthase
ISCs: Intestinal stem cells
IECs: Intestinal epithelial cells
BrdU: 5-Bromo-2′-deoxyuridine
EGF: Epidermal growth factor
Wnt3: Wingless-type MMTV integration site family member 3
QISCs: Quiescent intestinal stem cells
AISCs: Active intestinal stem cells
NO: Nitric oxide
NOSs: Nitric oxide synthases
eNOS: Endothelial nitric oxide synthase
nNOS: Neural nitric oxide synthase
Lgr5: Leucine-rich repeat-containing G-protein coupled receptor 5
EGFP: Enhanced green fluorescent protein
IRES: Internal ribosome entry site
PCR: Polymerase chain reaction
SPF: Specific pathogen-free
IHC: Immunohistochemistry
IF: Immunofluorescent
DEGs: Differentially expressed genes
GO: Gene Ontology
KEGG: Enrichment and Kyoto Encyclopedia of Genes and Genomes
GSEA: Gene set enrichment analysis
SD: Standard deviation
CBCs: Cryptal basal columnar cells
LPS: Lipopolysaccharides
TNF: Tumor necrosis factor
IGN: Intestinal gluconeogenesis
