# Supplementary material for: Paneth cell-derived iNOS is required to maintain homeostasis in the intestinal stem cell niche
Source: J Transl Med. 2023 Nov 25;21:852. doi: 10.1186/s12967-023-04744-w (PMC10675917; doi:10.1186/s12967-023-04744-w)
Supplement: Supplementary file 1 — Additional file 1: Figure S1. Phenotype characterization of iNOS−/− mice. A Genotyping of iNOS−/− mice was achieved by 1.5% agarose gel electrophoresis. Het = heterozygote, KO = knockout, NTC = no template control. B qPCR analysis of iNOS mRNA levels in wild-type mice and iNOS−/− mice. C Comparison of wild-type and iNOS−/− littermate body weights. *P < 0.05, n = 5. Figure S2. qPCR analysis of Nos2 in WT and iNOS−/− enteroids. Enteroids derived from the two groups were released and collected on day 4 after culturing, and then total RNA was extracted and used for qPCR testing. **P < 0.01. Figure S3. Loss of iNOS significantly decreased the protein level of p27 Kip1 in the intestinal crypts. Bar = 100 μm. Figure S4. Histogram of differentially expressed genes (DEGs) between the control group and iNOS−/− group. There were 168 upregulated genes (pink) and 155 downregulated genes (blue) in the iNOS−/− group compared with the control group. N = 3 for each group. Figure S5. qPCR validation for those genes implicated by RNA-Seq. A–D qPCR validation of the upregulated genes in RNA-Seq results. E–G qPCR validation of the genes that were downregulated in RNA-Seq. H qPCR results of the mRNA level of Dll4 gene, which was a component of Notch signaling pathway. I–L qPCR examination of adaptive immunity related genes. M–P qPCR validation of gluconeogenesis related genes. (*P < 0.05, ***P < 0.001). Figure S6. RNA-Seq data of inflammatory factors. The analysis of RNA-Seq data showed that mRNA levels of Tnf and Ifgn were significantly increased due to iNOS deficiency, but Il1b and Nfkb1 did not change significantly. (*P < 0.05). Figure S7. GSEA of genes downregulated in association with iNOS depletion. Differentially downregulated genes in the iNOS−/− group were also closely related to glycolytic processes in addition to gluconeogenesis (n = 3). [file 12967_2023_4744_MOESM1_ESM.docx]

**Supplementary figures and figure legends**


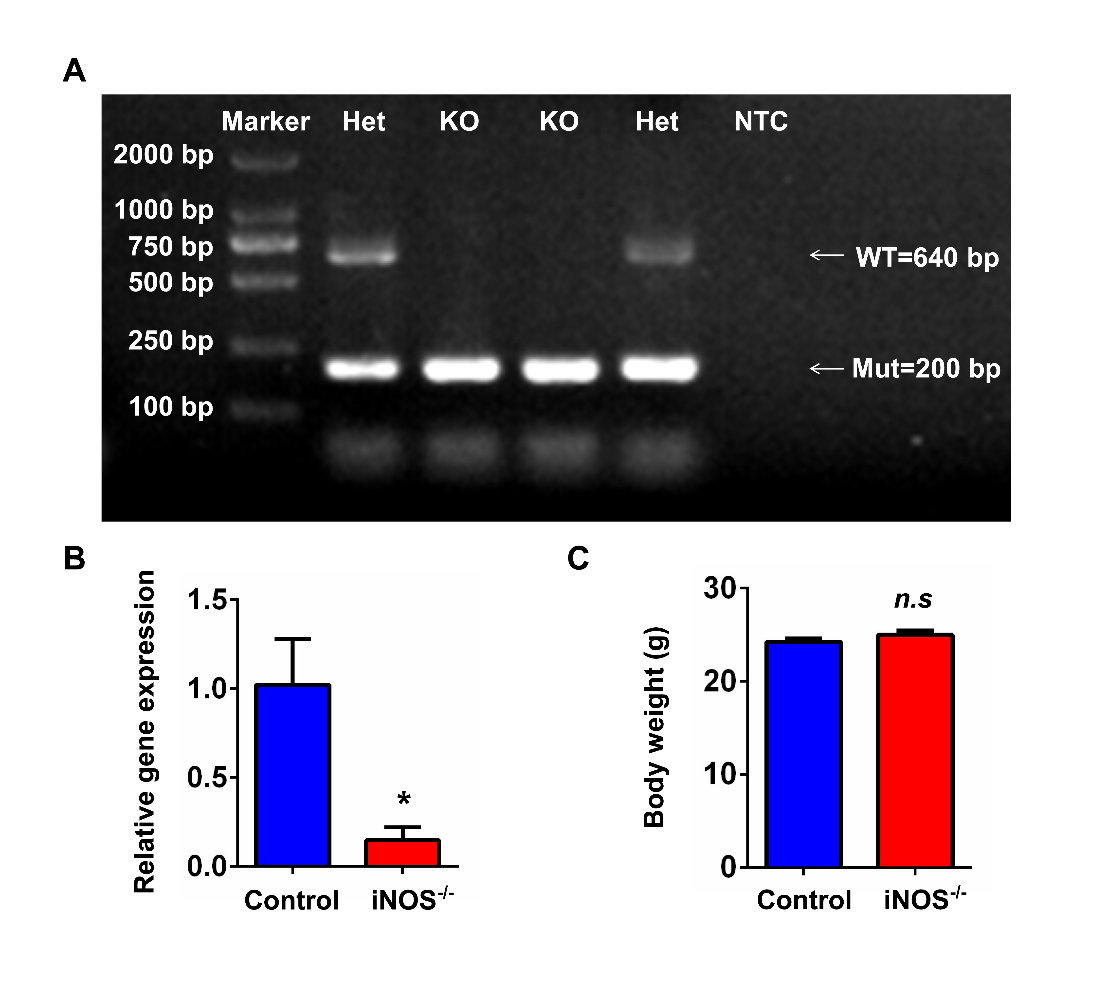


**Fig. S1 Phenotype characterization of iNOS^-/-^ mice.** **A** Genotyping of iNOS^-/-^ mice was achieved by 1.5% agarose gel electrophoresis. Het= heterozygote, KO= knockout, NTC= no template control. **B** qPCR analysis of iNOS mRNA levels in wild-type mice and iNOS^-/-^ mice. **C** Comparison of wild-type and iNOS^-/-^ littermate body weights. *: *P*< 0.05, n=5.


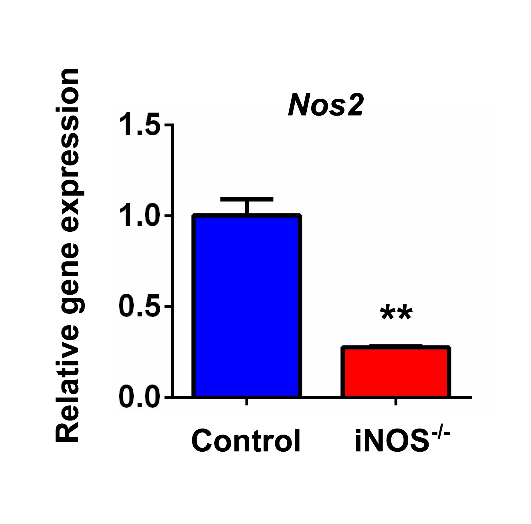


**Fig. S2 qPCR analysis of *Nos2* in WT and iNOS^-/-^ enteroids.** Enteroids derived from the two groups were released and collected on day 4 after culturing, and then total RNA was extracted and used for qPCR testing. **: *P*< 0.01.


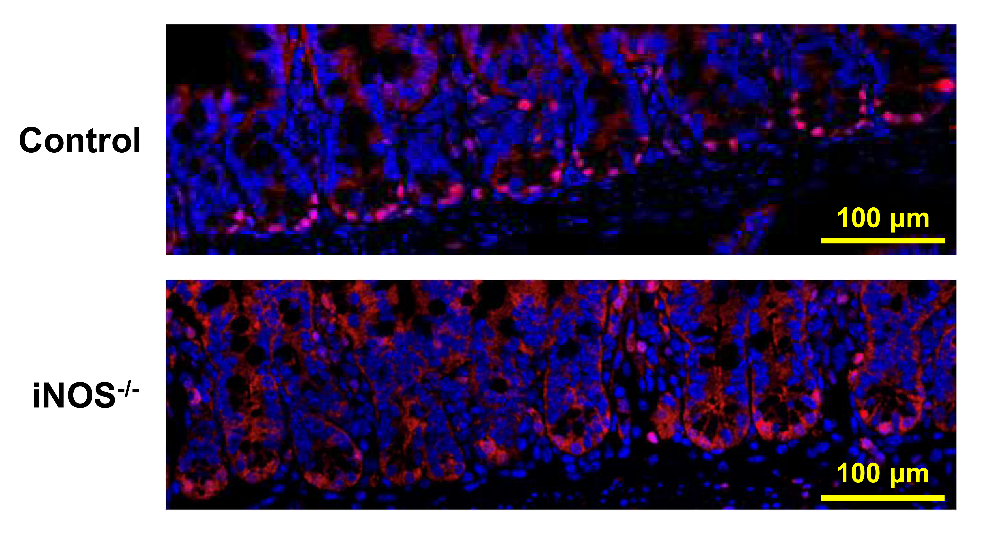


**Fig. S3 Loss of iNOS significantly decreased the protein level of p27 Kip1 in the intestinal crypts.** Bar= 100 μm.


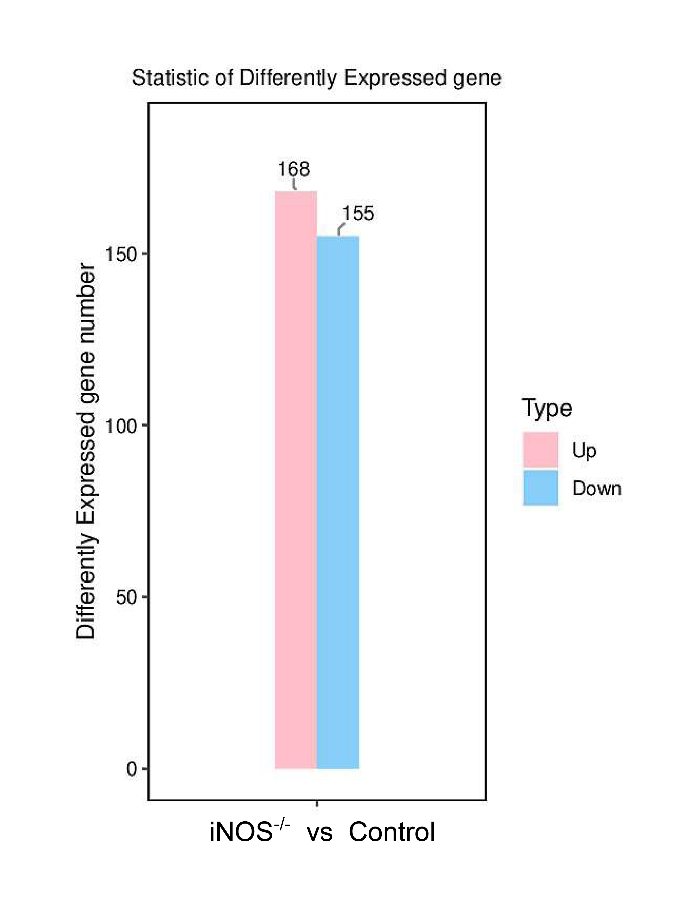


**Fig. S4 Histogram of differentially expressed genes (DEGs) between the control group and iNOS^-/-^ group.** There were 168 upregulated genes (pink) and 155 downregulated genes (blue) in the iNOS^-/-^ group compared with the control group. N=3 for each group.


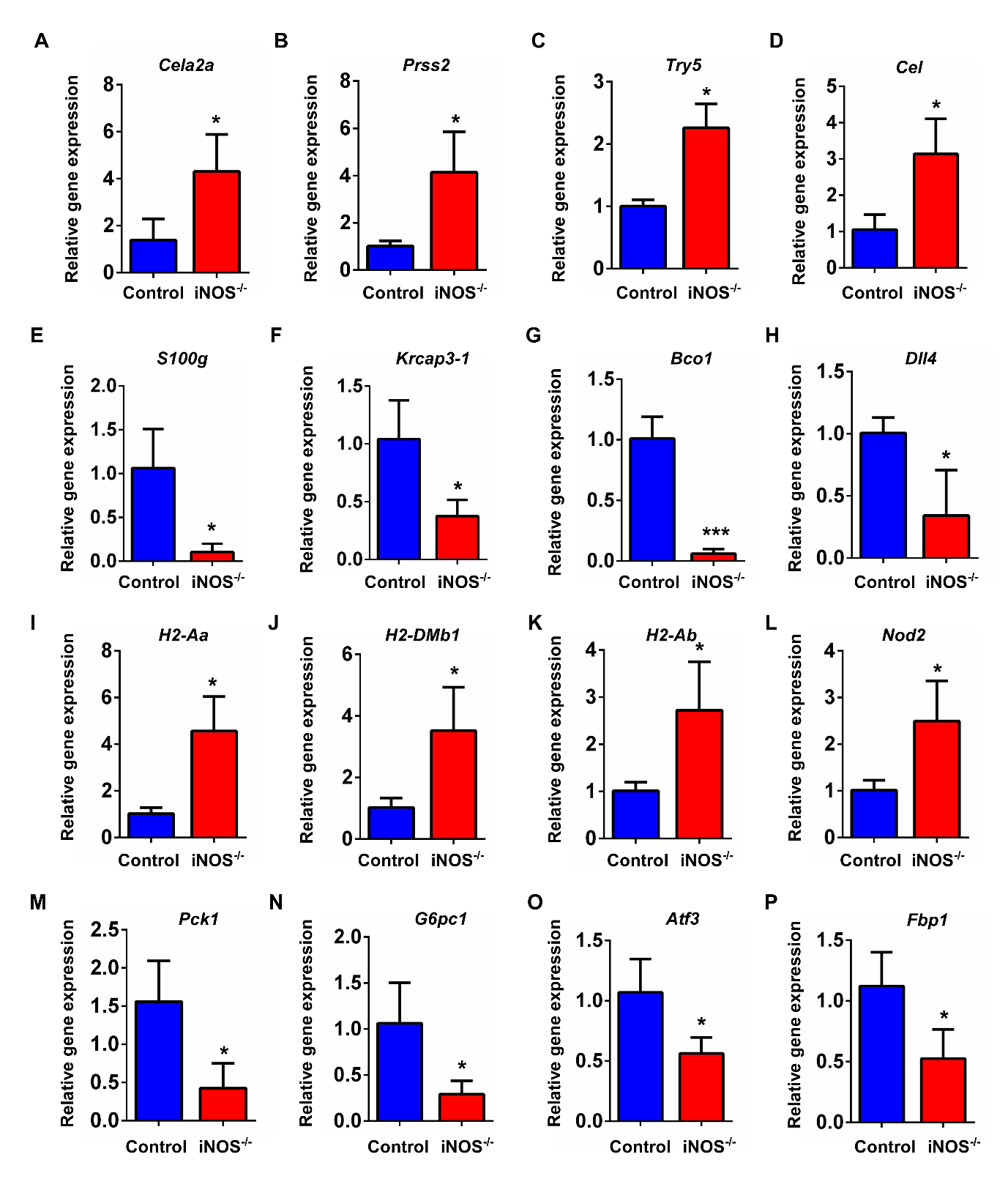


**Fig. S5 qPCR validation for those genes implicated by RNA-Seq.** A-D: qPCR validation of the upregulated genes in RNA-Seq results. E-G: qPCR validation of the genes that were downregulated in RNA-Seq. H: qPCR results of the mRNA level of Dll4 gene, which was a component of Notch signaling pathway. I-L: qPCR examination of adaptive immunity related genes. M-P: qPCR validation of gluconeogenesis related genes. (*: *P*<0.05, ***: *P*<0.001)


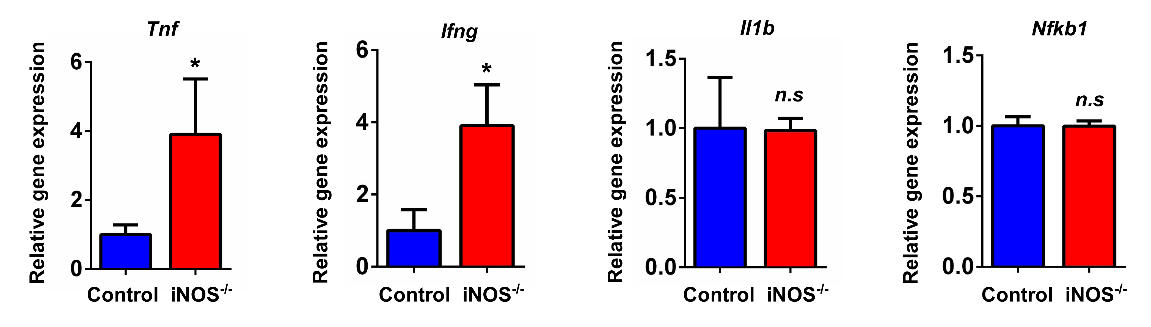


**Fig. S6 RNA-Seq data of inflammatory factors.** The analysis of RNA-Seq data showed that mRNA levels of Tnf and Ifgn were significantly increased due to iNOS deficiency, but Il1b and Nfkb1 did not change significantly. (*: *P*<0.05)


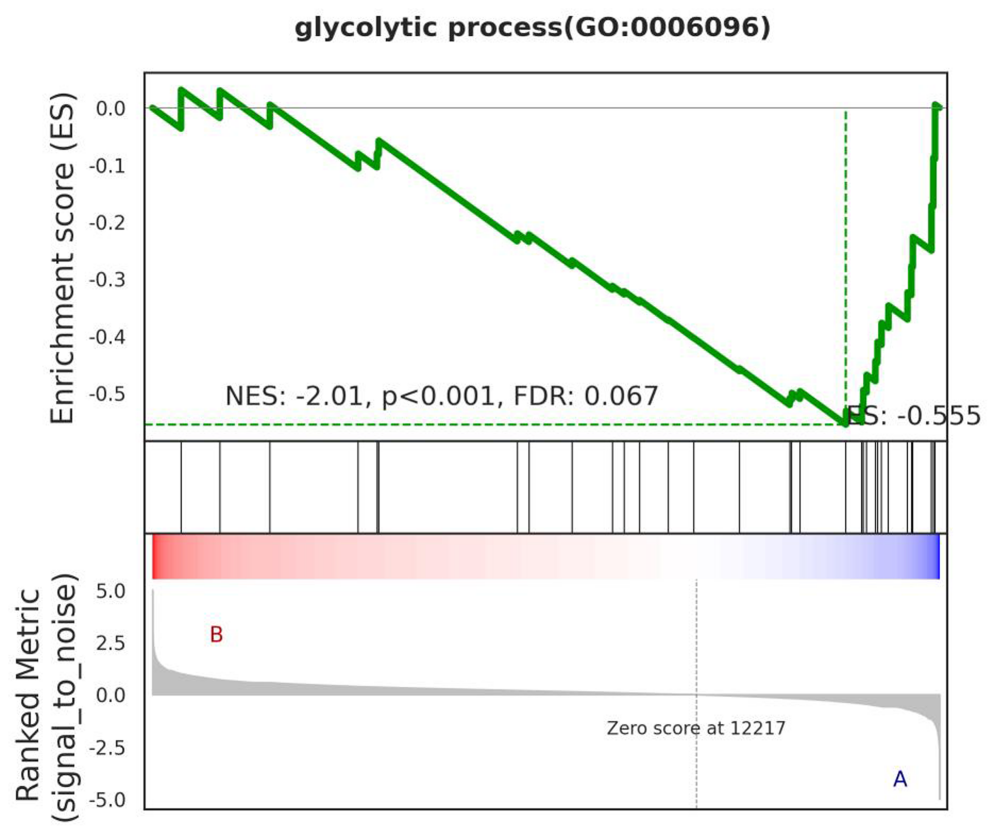


**Fig. S7 GSEA of genes downregulated in association with iNOS depletion.** Differentially downregulated genes in the iNOS^-/-^ group were also closely related to glycolytic processes in addition to gluconeogenesis (n=3).
